# The prevalence of symptoms and its correlation with sex in polish COVID-19 adult patients: Cross-sectional online open survey

**DOI:** 10.3389/fmed.2023.1121558

**Published:** 2023-04-05

**Authors:** Pawel Lewek, Izabela Banaś, Konrad Witkowski, Joanna Lewek, Przemyslaw Kardas

**Affiliations:** ^1^Department of Family Medicine, Medical University of Lodz, Łódź, Poland; ^2^Department of Preventive Cardiology and Lipidology, Chair of Nephrology and Hypertension, Medical University of Lodz, Łódź, Poland; ^3^Department of Cardiology and Adult Congenital Heart Diseases, Polish Mother's Memorial Hospital Research Institute (PMMHRI), Łódź, Poland

**Keywords:** COVID-19, symptoms, survey, questionnaire, sex correlation

## Abstract

**Background:**

The understanding and treatment of COVID-19 has improved rapidly since December 2019 when SARS-CoV-2 was sequenced. However most papers on its symptomatology focus on hospitalized patients and address only a limited number of major presentations. Although differences depending on sex of COVID-19 patients have been previously confirmed (higher ICU admission and higher death rate for men), no publication has focused on sex-related differences in COVID-19 symptomatology.

**Objective:**

The aim of the study was to present a reliable list of COVID-19 symptoms and identify any differences in symptom prevalence depending on sex.

**Methods:**

A sample of Polish patients suffering from COVID-19 were surveyed using a cross-sectional anonymous online survey in Polish available on a web-based surveying platform (Survey Monkey). The survey included 20 questions asking about COVID-19 symptoms, days of occurrence (from day 1 until day 14 and “15 days or more”) and patient characteristics including sex, age, height, weight, place of residence and type of therapy received during COVID-19. The survey was made available during the third COVID-19 wave in Poland. The link to the survey was distributed across social networks. Participation was open to anyone willing, without any incentives. The data was analyzed statistically.

**Results:**

Survey responses were collected from 2,408 participants (56.9% women) aged 18–90 (42 ± 12), 84.7% living in cities, who took part in the study between December 2020 and February 2021. Out of 54 predefined symptoms, the three most prevalent were fatigue (reported by 87.61% respondents), anosmia (73.74%) and headache (69.89%). Women were found to be more symptomatic than men, 31 symptoms occurred more often in women (including anosmia, headache and myalgias, *p* < 0.05). Subfebrility, fever and hemoptysis were more prevalent in men. Twelve symptoms (incl. hypothermia, sneezing and nausea) lasted longer in women than men (*p* < 0.05). Fatigue, cough, nasal dryness, xerostomia and polydipsia were the longest lasting symptoms of COVID-19 (lasted over 14 days).

**Conclusion:**

Our study presents a wide range of symptoms, which may enable better recognition of COVID-19, especially in an outpatient setting. Understanding these differences in the symptomatology of community and hospitalized patients may help diagnose and treat patients faster and more accurately. Our findings also confirmed differences in symptomatology of COVID-19 between men and women, which may lay the foundation for a better understanding of the different courses of this disease in the sexes. Further studies are necessary to understand whether a different presentation correlates with a different outcome.

## 1. Introduction

Severe acute respiratory syndrome coronavirus 2 (SARS-CoV-2) is a causative agent of COVID-19, which was discovered for the first time in December 2019 in Wuhan, China (1, 2), although COVID-19 is believed to have spread unnoticed throughout the Asian region much earlier (3). From this initial discovery, new cases soon began to be reported all over the world. The virus showed significant infectivity, reaching over 100 mln cases globally in one year (4). The first case in Poland was confirmed on March 4, 2021, and this was to rise to over 5.5 mln cases and over 100,000 deaths by the end of February 2022 (5).

Among the reported symptoms of COVID-19, the most common were fever (83–99%), headache (10–28%), cough (20–82%), fatigue (44–70%) and sore throat (14–19%) in adults (6, 7) and fever (46–64%) and cough (32–56%) in children (8). Although many papers have described COVID-19 manifestations, most have focused on selected symptoms (7), and to our knowledge, none have summarized all the possible signs of COVID-19 in a single population. Moreover most of the available studies only assess hospitalized patients (9) or fail to mention their origin (10). For example, one meta-analysis of COVID-19 symptoms (11) included research from 33 articles conducted on 15,244 hospitalized patients and only 9,011 non-hospitalized patients; in addition, these present only limited number of symptoms, ranging from 13 (12) to 23 (13). Another metanalysis included 24,410 hospitalized patients from four continents; however, no data was included from Central or Eastern Europe (14). While the paper presents a high number of symptoms (n = 30), the authors concede that clear differences exist between countries, and this prevents generalization of findings (14). As such, studies are needed on single populations. While it is important to present individual symptoms, such studies run the risk of ignoring the full picture of the disease, and becoming restricted to the most common cases.

Moreover publications based on hospital rather than community settings may upwardly bias the estimates of symptom prevalence, and not reflect the true numbers in the general population (14). Hospitalized patients also differ significantly from the general population in terms of disease severity, aggravation of symptoms and type of care received. In addition, while most COVID-19 patients are consulted in primary care, which uses different decision making processes from hospitals, most current publications on hospitalized patients focus on major symptoms and lab tests. Therefore it is crucial to have a full overview of all possible symptoms of COVID-19 to allow precise diagnosis; this is particularly true for primary care physicians. Other publications on COVID-19 symptoms have examined population type and ethnicity (15), machine learning (16), mobile applications (17) and social media (18, 19).

In addition to symptoms and clinical presentation, the influence of sex on COVID-19 manifestation is of great interest. It was found soon after the outbreak of epidemic that the course of the disease was depended on the sex of the patient. For example, 73% of the 116 deceased COVID-19 patients from Wuhan Tongji Hospital in China in January–February 2020 were men (20). Data from South Korea also showed a 2:1 female to male ratio of COVID-19 cases, but with twice the mortality among men (21). A report from the Italian National Institute of Health from April 2020 stated that approximately 70% of COVID-19 deaths were men, while the National Centre for Health Statistics in the US reported that 59% out of 37,308 deaths were men (22). Another study found the case fatality ratio for men to women to be 1.7 in Italy, Spain and Sweden and 1.4 in Germany (23). Finally, a meta-analysis of over 3.1 million COVID-19 cases confirmed that despite there being no sex-related difference in the chance of being infected with COVID-19, men were three times more likely to require treatment in the intensive care unit and to have a higher chance death (24).

Despite this, to date, no studies appear to have examined differences in COVID-19 symptoms according to sex. Moreover, no individual publication has examined the vast collection of known COVID-19 symptoms in a single population, including non-hospitalized patients. Therefore, the aim of this article is to provide an overview of the symptomatology of COVID-19, and to support physicians, especially those of outpatients, in the diagnostic process.

## 2. Aim

The primary aim of the study was to build a reliable list of possible COVID-19 symptoms based on a survey of COVID-19 positive patients about the symptoms experienced during the first 14 days of infection. The secondary aim was to identify sex-based differences in symptom prevalence.

## 3. Methods

### 3.1. Recruitment

A cross-sectional study was performed using a bespoke questionnaire based on the previous experience of the authors and a literature review of COVID-19 cases and reported symptoms. The survey was made available in Polish through a web based surveying platform (Survey Monkey) during the 3rd COVID-19 wave in Poland, between December 23, 2020 and February 28, 2021. The invitation to the survey was distributed across social networks including related Facebook groups and the commentary fields of news web sites devoted to the topic of COVID-19 (Supplementary material S1). Participation in the study was voluntary. The link to the survey was not password protected and hence was open to anyone who was willing to complete it. (The survey announcement is presented in Supplementary material S2). The survey was completely anonymous, IP numbers were not collected, cookies were not used to assign any unique user identifier to each client computer. No incentives (monetary or non-monetary) were offered for participation in the survey.

### 3.2. Survey design

A cross-sectional online open survey was performed on a convenience sample of Polish-speaking individuals with internet access who were willing to take part in the study.

The first question asked about previous participation in the survey: if answered “yes,” the participant was not allowed to continue. It also asked whether the respondent was completing the survey on behalf of other person (e.g., elderly, child), which was allowed. Authenticity of the answer was not verified otherwise, it was based on patient’s declaration.

The initial screen included a welcome message, introduction about the authors of the survey, explanation of study purpose, information about anonymization and possibility to withdraw at any stage, a request to give sincere and honest answers, as well as ethical board approval and lack of sponsorship of any kind. Participants were also informed about the time required to fill in the survey and given basic information on how to navigate the survey.

The second screen included two questions about exclusion criteria. To be included in the final analysis, the participants had to meet the following criteria: no previous participation in the survey, self-reported positive RT-PCR test confirming SARS-CoV-2 infection, answer all questions about the experienced COVID-19 symptoms. The survey included 20 questions (only selected questions were used for the following analysis) and was presented on five pages with two to nine questions on each page. All questions were available for every participant (no adaptive questioning was applied). Answering every question was mandatory and the participant was alerted if a question was missed.

On the third screen, the participants were presented with a list of 54 pre-defined symptoms, and were asked to indicate which ones they had experiences, and on which days of the illness (day 1 to 14 or “day 15 and beyond”). A “no symptoms” option was also available. One of the questions included a list of COVID-19 symptoms based on available publications (25–28), authors’ clinical experience, and symptoms additionally proposed by participants during the pilot stage of the study. The symptoms were not defined or explained (except temperature and skin lesions, see below), but were self-explanatory, and written in lay language (e.g., ‘lack of smell’ instead of ‘anosmia’). The body temperature was divided into ‘lowered temperature’ (hypothermia, defined in a survey as temperature 36,1 deg.C or lower), subfebrility (37.0–37.9 Centigrades) and fever (≥38.0 deg.C). Although this division is not common in English literature, it is widely used and understood by Polish patients and as such was proposed in the survey. This followed the understanding of normal axillary temperature range of 36.2–36.9 Centigrade and fever definitions given in the literature (29, 30). Skin lesions were defined in the survey by adding an example ‘e.g. rash, pimples’. Our findings were reported according to the CHERRIES checklist for on-line survey reporting (31).

Until the final submission, participants were able to go back and change any answer to any question. The contact details to the principal investigator were provided at the final screen of the survey.

### 3.3. Completion and completeness rate

Completion rate (ratio of users who finished the survey/users who agreed to participate) and completeness rate (measure for how completely questionnaires were filled, without questionnaires left blank) were counted after results were obtained. Questionnaires completed partially (i.e., not to the end) were included in the final analysis; however, the numbers of participants differed depending on the question. While it was obligatory to answer all questions, the participant was able to quit at any point. The answers were registered to the last question that had been answered. Responses to the question were recorded when respondent clicked the navigation buttons in the survey (e.g., ‘next’ button), therefore only fully completed questions were included in the final analysis.

### 3.4. Statistical analysis

The Shapiro–Wilk test was conducted to check the normality of the data distribution. Some variables were converted into categorical variables: BMI, city of residence, stated needs for diverse types of therapy during COVID-19 infection. For continuous variables, the obtained results were reported as mean ± standard deviation for Gaussian distribution or median with 25–75% percentiles if not. Discrete variables were presented as proportions. Comparisons between groups were conducted with the Student’s t-test for independent variables, the Mann–Whitney U test or χ2 test with Yates correction and Kruskal-Wallis test, as appropriate. For all calculations, *p*-values <0.05 were considered statistically significant. The statistical analysis was performed using STATISTICA v.13 software (TIBCO Software Inc., Palo Alto, CA, USA).

### 3.5. Pilot

The preliminary version of the questionnaire was piloted on a group of seven family physicians and 10 patients in order to check for errors, ensure that the survey was clear and to generally polish it.

#### 3.5.1. Ethics approval

The study design was reviewed by the ethical commission as research involving human subjects. According to decision no. RNN/319/20/KE, December 15, 2020 of the Bioethical Commission of the Medical University of Lodz, the study protocol and survey design was approved as an observational study. The participant was informed that by joining and starting the survey, he or she was giving their informed consent to complete the survey anonymously, and that he or she accepted that the data entered may be used on the basis of this informed consent for collective data analysis without additional consent. All data was collected anonymously, thus privacy and confidentiality of participants was adequately protected. Participants were informed that filling in the questionnaire is completely voluntary, and no compensation financial or otherwise was provided.

## 4. Results

**Figure 1:**
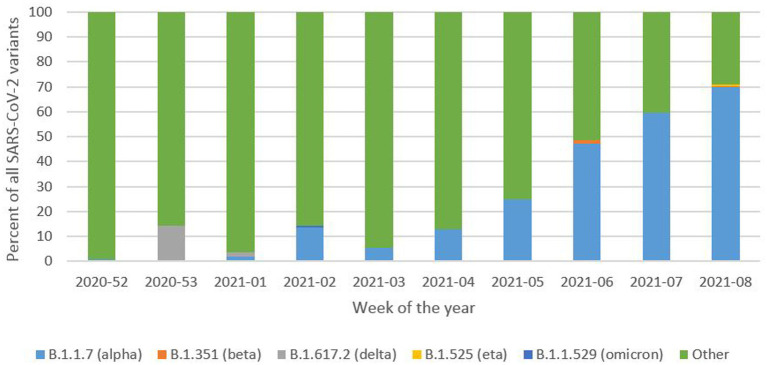
SARS-CoV-2 variant distribution in Poland reported during the time of survey response collection. Based on the data available from: (32).

The survey responses were collected between December 23, 2020 (week 52 of 2020) and February 28, 2021 (week 08 of 2021) during the dominance of SARS-CoV-2 alpha variant (Figure 1) (32). A total number of 7,034 participants started the survey. All confirmed that they were taking part in the survey for the first time or writing the survey on behalf of other person (child, elderly).

The completion rate was 1783/7034*100% = 25.25%. Completeness was 1740/7034*100% = 24.74%. In total, 1740 participants answered all questions. As partially-completed questionnaires were included in the final analysis, the number of participants is presented with each result. In general, the later questions in the survey elicited fewer responses.

**Figure 2:**
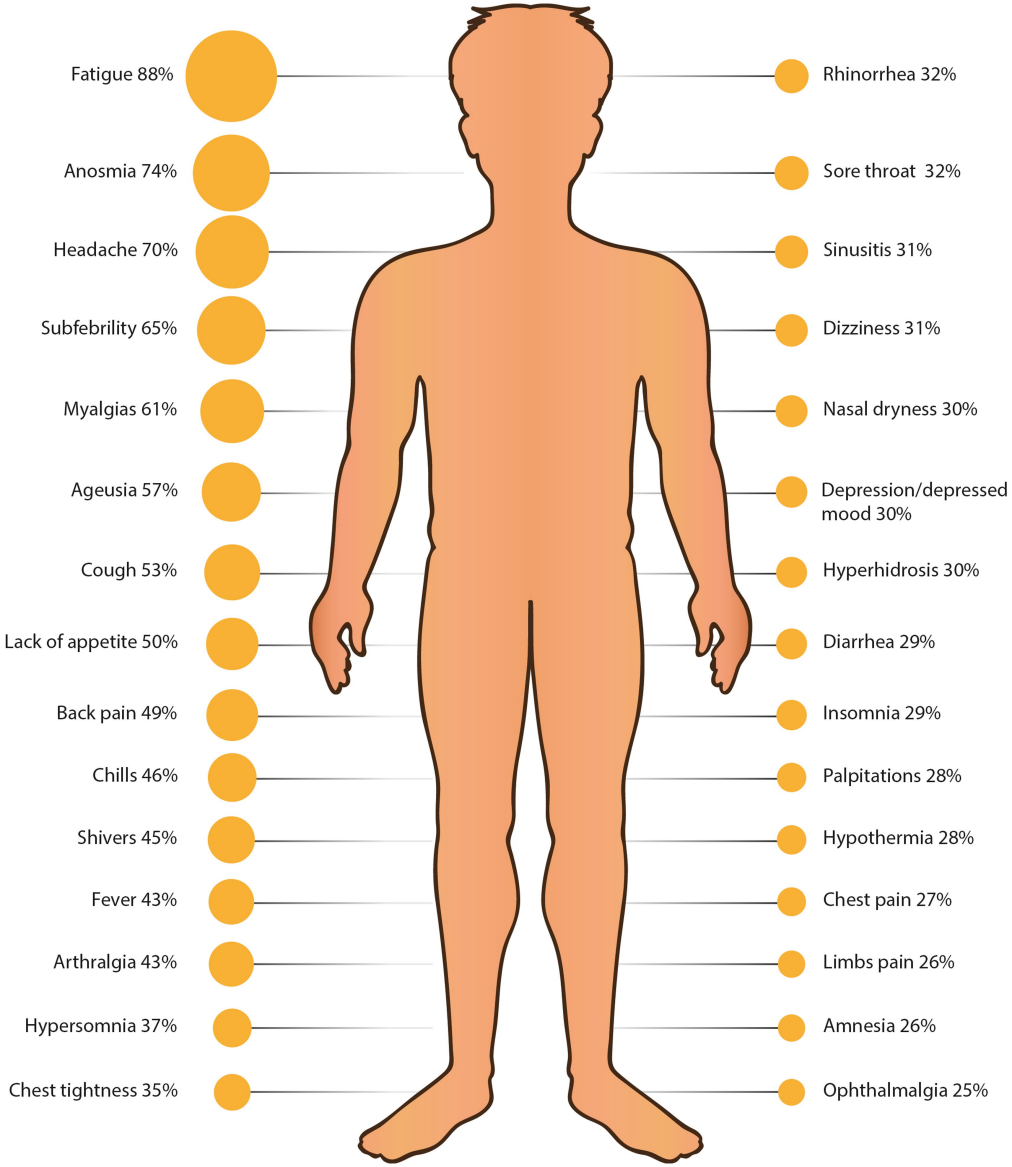
Thirty most common COVID-19 symptoms reported by survey participants. Man figure source: freepik.com.

Some questionnaires were excluded for the following reasons: participants claimed that they had answered the survey previously (80 surveys excluded), participants reported that they have not been tested for SARS-CoV-2 (*n* = 2,579), participants were younger than 18 years (*n* = 15), no answer was given to questions about symptoms (or question about ‘lack of symptoms’) (*n* = 1951) or duplicates of the same answer were found (*n* = 1). Therefore, 2,408 surveys were included in the final analysis, representing 2,408 patients (397 men −16.49% and 1,370 women—56.89%, 641 no data given—26.62%) with an age range from 18 to 90 years.

The characteristics of the participants are provided in Table 1. Most of the participating men were overweight while most women were of normal weight (Table 2). Most participants were living cities with populations of 10,001 to 100,000 inhabitants (Table 2) and did not require hospitalization, oxygen therapy, respiratory therapy or a stay in the isolation unit (Table 2).

**Table 1:** Characteristics of studied population (*N* = 397 men, *N* = 1,370 women).

	Median (LQ-UQ) (men)	(Men) Mean ± SD	Median (LQ-UQ) (women)	(Women) Mean ± SD	*p*	Median (LQ-UQ) total	Total Mean ± SD
Age (years)	41 (34–52)	42 ± 12	42 (34–50)	42 ± 12	0.592	42 (34–50)	42 ± 12
Height (cm)	180 (175–185)	180 ± 7	167 (162–170)	166 ± 8	<0.001	168 (164–175)	169 ± 9
Weight (kg)	88 (78–97)	89 ± 17	68 (60–80)	71 ± 15	<0.001	72 (62–85)	75 ± 17
Body mass index (kg/m^2^)	26.87 (24.34–29.73)	27.59 ± 4.94	24.75 (21.71–28.58)	25.64 ± 5.36	<0.001	25.31 (22.31–29.05)	26.08 ± 5.33

**Table 2:** Body mass, place of residence, and needs of therapy among studied population.

**Body mass characteristics of studied population (*p* < 0.001)**						
Body mass index category (range in kg/m^2^)*	n total	n men	n women	%	% men	% women
Normal (18.5 to <25.0)	799	126	673	45.22	31.74	49.12
Underweight (<18.5)	43	2	41	2.43	0.50	2.99
Overweight (25.0 to <30.0)	575	178	397	32.54	44.17	28.81
Obese I (30.0 to <35.0)	232	63	169	13.13	15.63	12.26
Obese II (35.0 to <40.0)	86	19	67	4.87	4.71	4.86
Obese III (≥40.0)	32	9	23	1.81	2.23	1.67
**Place of residence of studied population (*p* = 0.55)**						
City with more than 1 mln inhabitants	342	76	266	19.35%	19.14%	19.42%
City from 500.001 to 1 mln inhabitants	296	74	222	16.53%	18.64%	16.20%
City from 100.001 to 500.000 inhabitants	352	83	269	19.92%	20.91%	19.64%
City from 10.001 to 100.000 inhabitants	423	82	341	23.94%	20.65%	24.89%
City up to 100.000 inhabitants	84	21	63	4.75%	5.29%	4.60%
Rural area	270	61	209	15.28%	15.37%	15.26%
**Stated needs for diverse types of therapy during COVID-19 infection**						
Stay at the hospital	84	35	49	3.49%	41.67%	58.33%
Oxygen therapy	73	29	44	3.03%	39.73%	60.27%
Respirator application	6	3	3	0.25%	50.00%	50.00%
Stay in isolation unit	22	12	10	0.91%	54.55%	45.45%
None of the above	1,653	356	1,297	68.65%	21.54%	78.46%

* BMI range based on (31).

Among all the respondents, the three most prevalent symptoms were fatigue (87.61%), anosmia (73.74%) and headache (69.89%) (Figure 2, Table 3). These symptoms were present in most patients on day 5 (67.32% of surveyed patients), day 7 (56.64%) and day 2 (46.14%) respectively (Figure 5). Most patients reported fatigue (58.07%), headache (44.12%) and myalgias (37.39%) on the first day (Figure 5).

**Table 3:** Prevalence of symptoms and their duration in studied population (*N* = 2,408).

No.	Symptom	n	Percentage (%)	Duration of the symptom in days (median (LQ-UQ))	Minimum duration in days	Maximum duration in days	Duration of the symptom in days average (± SD)	Mode in days
1	Fatigue	1,548	87.61	11 (6–15)	1	15	9.88 (±3.19)	15
2	Anosmia	1,303	73.74	9 (6–12)	1	15	8.5 1(±3.35)	10
3	Headache	1,235	69.89	5 (3–9)	1	15	6.03 (±4.18)	3
4	Subfebrility	1,154	65.31	3 (2–5)	1	15	4.21 (±4.72)	2
5	Myalgias	1,077	60.95	5 (3–8)	1	15	5.61 (±3.93)	3
6	Ageusia	1,002	56.71	8 (5–11)	1	15	8.11 (±4.09)	7
7	Cough	942	53.31	8 (4–13)	1	15	8.28 (±4.26)	15
8	Lack of appetite	885	50.08	8 (5–11)	1	15	7.92 (±4.09)	5
9	Back pain	873	49.41	5 (3–8)	1	15	5.75 (±3.97)	3
10	Chills	820	46.41	4 (2–7)	1	15	5.01 (±4.00)	2
11	Shivers	796	45.05	3 (2–6)	1	15	4.28 (±4.47)	2
12	Fever	768	43.46	3 (2–5)	1	15	3.95 (±3.64)	1
13	Arthralgia	764	43.24	5 (2–8)	1	15	5.59 (±3.85)	1
14	Hypersomnia	653	36.96	7 (4–11)	1	15	7.46 (±3.17)	3.7
15	Chest tightness	617	34.92	5 (2–8)	1	15	5.55 (±3.17)	1
16	Rhinorrhea	574	32.48	5 (3–8)	1	15	5.96 (±3.78)	3
17	Sore throat	573	32.43	3 (2–5)	1	15	4.25 (±4.00)	3
18	Sinusitis	556	31.47	5 (3–8)	1	15	6.06 (±3.96)	3
19	Dizziness	545	30.84	5 (3–8)	1	15	6.11 (±4.65)	1
20	Nasal dryness	536	30.33	7 (4–111)	1	15	7.79 (±4.10)	15
21	Depression/depressed mood	529	29.94	7 (4–11)	1	15	7.36 (±4.41)	1
22	Hyperhidrosis	522	29.54	7 (4–10)	1	15	7.14 (±3.15)	5
23	Diarrhea	521	29.49	3 (1–5)	1	15	3.67 (±4.42)	1
24	Insomnia	516	29.2	6 (3–10)	1	15	6.84 (±3.75)	1
25	Palpitations	498	28.18	5 (3–9)	1	15	6.20 (±3.78)	1
26	Hypothermia	496	28.07	4 (2–8)	1	15	5.6 (±2.42)	1
27	Chest pain	484	27.39	5 (3–9)	1	15	5.84 (±3.09)	1
28	Limbs pain	460	26.03	5 (3–8)	1	15	6.06 (±3.95)	3
29	Amnesia	455	25.75	4 (1–8)	1	15	5.35 (±4.02)	1
30	Ophthalmalgia	450	25.47	5 (3–7)	1	14	5.44 (±3.90)	3
31	Dyspnoea	411	23.26	5 (3–8)	1	15	5.96 (±4.12)	1
32	Xerostomia	404	22.86	7 (4–12)	1	15	7.96 (±3.92)	15
33	Diaphoresis	372	21.05	5 (3–8)	1	15	5.69 (±2.90)	4
34	Nausea	365	20.66	4 (2–7)	1	15	5.26 (±4.12)	1
35	Sneezing	351	19.86	4 (2–7)	1	15	5.13 (±4.13)	3
36	Abdominal pain/ache	347	19.64	3 (2–6)	1	15	4.61 (±4.32)	1
37	Hoarseness	339	19.19	5 (2–9)	1	15	5.98 (±4.16)	1
38	Shoulder pain	336	19.02	5 (3–8)	1	15	6.00 (±4.51)	3
39	Skin hyperaesthesia	323	18.28	5 (3–7)	1	15	5.57 (±4.28)	3
40	Polydipsia	308	17.43	8 (5–12)	1	15	8.05 (±4.17)	15
41	Alopecia	274	15.51	4 (1–8)	1	13	5.31 (±3.99)	1
42	Metallic taste	272	15.39	5 (3–8)	1	14	5.97 (±4.58)	1
43	Limb numbness	225	12.73	4 (1–8)	1	15	5.37 (±3.50)	1
44	Skin lesions	187	10.58	4 (2–7)	1	15	4.82 (±3.88)	1
45	Conjunctivitis	187	10.58	4 (2–7)	1	15	5.33 (±4.00)	1
46	Ear ache/pain	171	9.68	3 (1–5)	1	15	3.65 (±4.28)	1
47	Blood pressure elevation	168	9.51	5 (3–8)	1	15	5.97 (±4.14)	3
48	Odontalgia	139	7.87	4 (2–6)	1	15	4.51 (±4.58)	1
49	Vomiting	125	7.07	2 (1–4)	1	15	2.82 (±4.31)	1
50	Blood pressure reduction	99	5.6	6 (2–10)	1	15	6.33 (±4.33)	1
51	Urinary incontinence	61	3.45	5 (2–10)	1	13	5.72 (±3.49)	1
52	Aphonia	47	2.66	2 (1–5)	1	15	3.53 (±3.57)	1
53	Toes discoloration	38	2.15	2 (1–8)	1	15	4.47 (±4.38)	1
54	Hemoptysis	37	2.09	2 (1–5)	1	15	3.24 (±3.96)	1
	Total	2,408	100.00					

**Figure 3:**
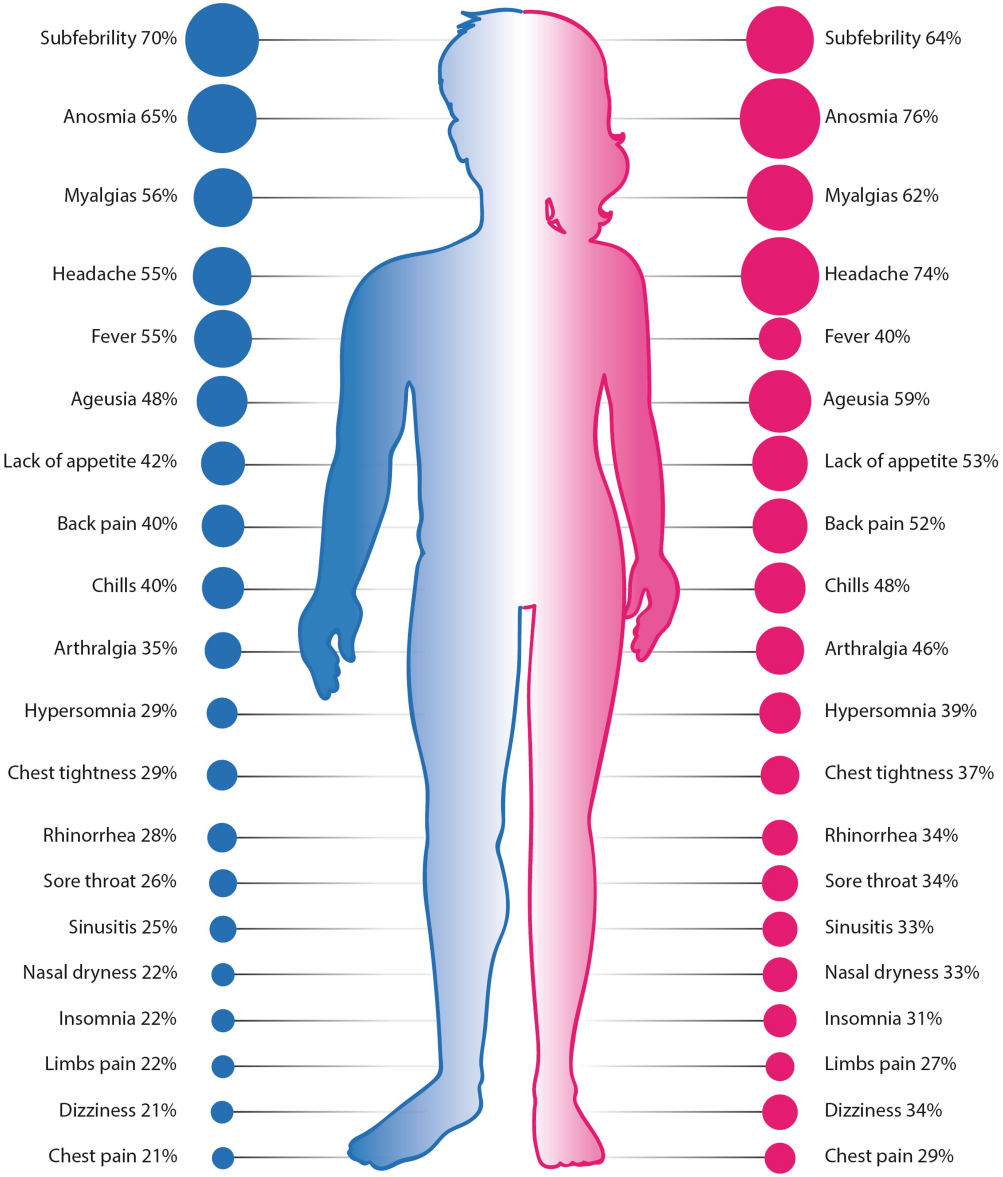
Comparison of 20 most common symptoms reported by men and women. Differences between all presented symptoms were statistically significant (*p* < 0.05). Source of figures: freepik.com.

**Figure 4:**
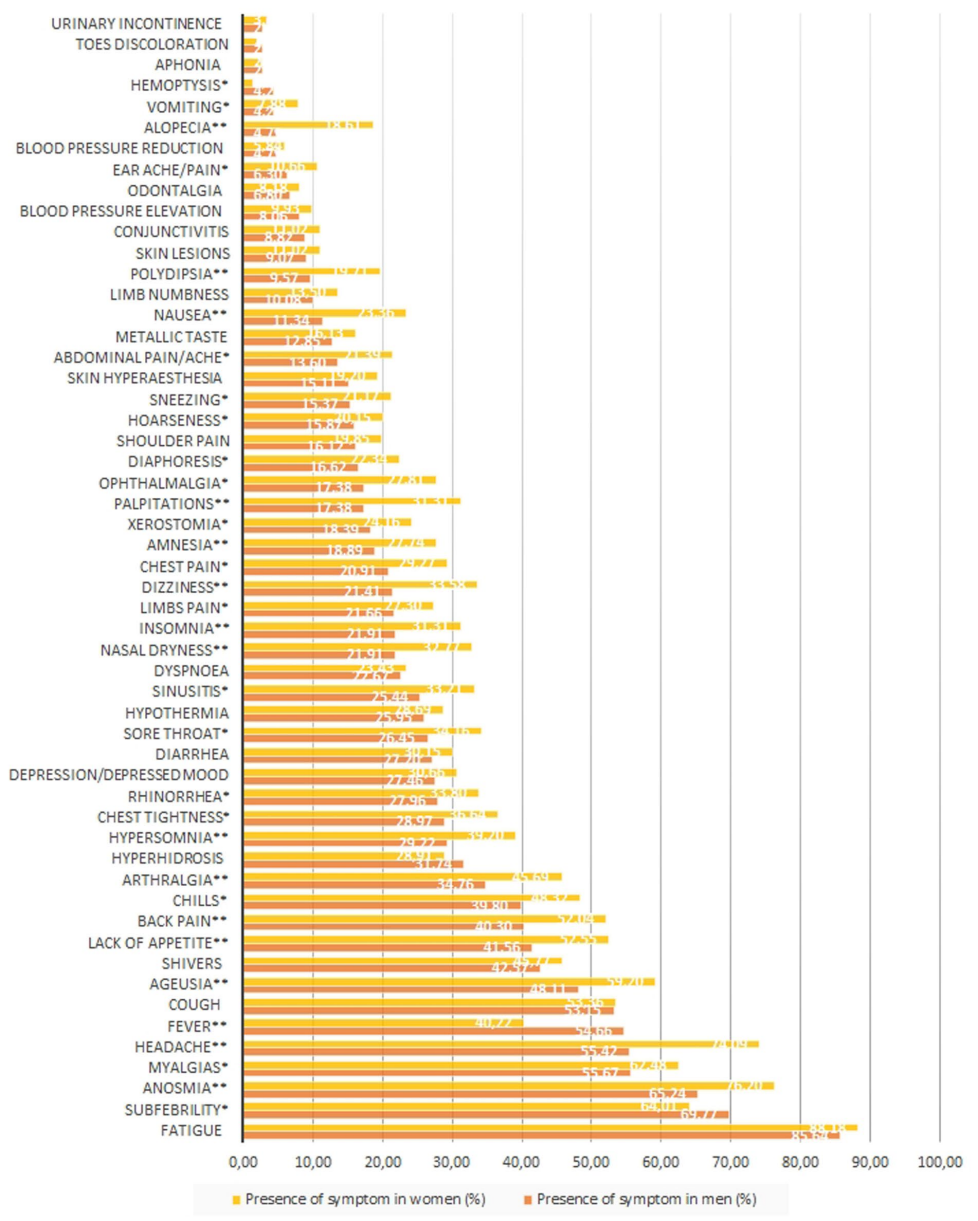
Prevalence of COVID-19 symptoms among men and women in studied population. *p<0.05, **p<0.005.

Statistically significant differences in the prevalence of COVID-19 symptoms were found between man and women (Figure 3). Women were generally more symptomatic than men, with 31 symptoms occurred significantly more often in women than in men; their frequencies in descending order were as follows (as given in Table 4 and Figure 4): anosmia (reported by 76% of women), headache (74%), myalgias (62%), lack of appetite (52%), back pain (52%), chills (48%), arthralgias (45%), hyperosmia (39%), chest tightness (36%), rhinorrhea (34%), sore throat (34%), dizziness (33%), nasal dryness (33%), sinusitis (33%), insomnia (31%), palpitations (31%), chest pain (29%), amnesia (28%), ophthalmalgia (28%), limbs pain (27%), xerostomia (24%), nausea (23%), diaphoresis (22%), abdominal pain/ache (21%), sneezing (21%), hoarseness (20%), polydipsia (20%), alopecia (19%), ear pain/ache (11%) and vomiting (8%). Only three symptoms were more prevalent in men: subfebrility (reported by 70% of men), fever (55%) and hemoptysis (4%).

**Table 4:** Sex differences in COVID-19 manifestation [*N* = 1767].

Symptom	Presence of symptom in men (%)	Presence of symptom in women (%)	*p*	Duration of symptom in men^1^	Min	Max	Avg^3^	Mode	Duration of symptom in women^2^	Min	Max	Avg^3^	Mode	*p*
Fatigue	85.64	88.18	0.177	10 (5–14)	1	15	9.34 (±4.65)	15	11 (6–15)	1	15	10.02 (±4.73)	15	0.007
Subfebrility	69.77	64.01	0.034	3 (2–5)	1	15	3.97 (±3.17)	1	3 (2–5)	1	15	4.29 (±3.40)	2	0.183
Anosmia	65.24	76.20	0.000	8 (6–12)	1	15	8.41 (±3.98)	6	9 (6–12)	1	15	8.54 (±4.01)	10	0.600
Myalgias	55.67	62.48	0.014	5 (3–8)	1	15	5.71 (±3.71)	1	4 (3–8)	1	15	5.58 (±3.98)	3	0.252
Headache	55.42	74.09	0.000	5 (3–8)	1	15	5.68 (±4.06)	3	5 (3–9)	1	15	6.11 (±4.30)	3	0.222
Fever	54.66	40.22	0.000	3 (2–6)	1	15	4.26 (±3.37)	1	3 (2–5)	1	15	3.83 (±3.11)	2	0.138
Cough	53.15	53.36	0.941	8 (4–12)	1	15	8.15 (±4.62)	15	8 (4–13)	1	15	8.32 (±4.66)	15	0.663
Ageusia	48.11	59.20	0.000	7 (5–11)	1	15	7.91 (±3.95)	7	8 (5–11)	1	15	8.15 (±3.96)	10	0.418
Shivers	42.57	45.77	0.260	3 (2–6)	1	14	3.98 (±3.04)	2	3 (2–6)	1	15	4.36 (±3.20)	2,3	0.074
Lack of appetite	41.56	52.55	0.000	6 (4–10)	1	15	7.20 (±4.22)	5	8 (5–11)	1	15	8.08 (±3.96)	7	0.007
Back pain	40.30	52.04	0.000	4 (2–7)	1	15	5.40 (±3.96)	1	5 (3–8)	1	15	5.82 (±4.01)	3	0.154
Chills	39.80	48.32	0.003	4 (2–6)	1	15	4.68 (±3.55)	1	4 (2–7)	1	15	5.08 (±3.84)	2	0.242
Arthralgia	34.76	45.69	0.000	5 (3–8)	1	15	5.88 (±4.14)	1	4 (2–8)	1	15	5.53 (±4.08)	1	0.295
Hyperhidrosis	31.74	28.91	0.276	6 (3–9)	1	15	6.31 (±4.10)	1	7 (4–10)	1	15	7.41 (±4.10)	5	0.008
Hypersomnia	29.22	39.20	0.000	6 (4–10)	1	15	7.22 (±4.36)	3,4,7	7 (4–11)	1	15	7.52 (±4.27)	3,7	0.414
Chest tightness	28.97	36.64	0.005	5 (2–8)	1	15	5.23 (±3.65)	1	5 (2–8)	1	15	5.62 (±4.04)	1	0.542
Rhinorrhea	27.96	33.80	0.029	5 (3–9)	1	15	6.20 (±4.26)	3	5 (3–8)	1	15	5.91 (±4.07)	3	0.567
Depression/depressed mood	27.46	30.66	0.220	7 (4–11)	1	15	7.37 (±4.56)	1	7 (4–11)	1	15	7.35 (±4.59)	1	0.978
Diarrhea	27.2	30.15	0.258	3 (1–4.5)	1	15	3.61 (±3.03)	1	3 (1–5)	1	15	3.68 (±3.11)	1	0.966
Sore throat	26.45	34.16	0.004	3 (2–5)	1	15	4.50 (±3.75)	3	3 (2–5)	1	15	4.20 (±3.40)	3	0.529
Hypothermia	25.95	28.69	0.284	3 (2–6)	1	15	4.54 (±3.80)	1	5 (3–8)	1	15	5.88 (±4.24)	3	0.001
Sinusitis	25.44	33.21	0.003	5 (3–7)	1	15	5.56 (±3.57)	3	5 (3–8)	1	15	6.18 (±3.90)	3	0.157
Dyspnoea	22.67	23.43	0.752	5 (3–9)	1	15	6.26 (±4.30)	1	5 (3–8)	1	15	5.87 (±3.79)	3	0.667
Nasal dryness	21.91	32.77	0.000	7 (3–11)	1	15	7.22 (±4.50)	1	7 (5–11)	1	15	7.90 (±4.40)	15	0.186
Insomnia	21.91	31.31	0.000	5 (2–8)	1	15	6.00 (±4.31)	1	6 (3–10)	1	15	7.01 (±4.54)	15	0.053
Limbs pain	21.66	27.30	0.024	5 (3–9)	1	15	5.91 (±3.89)	3,5	5 (3–8)	1	15	6.10 (±4.03)	3	0.755
Dizziness	21.41	33.58	0.000	5 (2–8)	1	15	5.96 (±4.23)	1	5 (3–8)	1	15	6.14 (±4.13)	1	0.625
Chest pain	20.91	29.27	0.001	5 (2–8)	1	15	5.77 (±4.24)	1	5 (3–9)	1	15	5.86 (±4.06)	1	0.708
Amnesia	18.89	27.74	0.000	4 (1–7)	1	15	5.00 (±4.34)	1	5 (1–8)	1	15	5.42 (±4.13)	1	0.298
Xerostomia	18.39	24.16	0.016	6 (3–10)	1	15	6.92 (±4.42)	1	8 (4–12)	1	15	8.19 (±4.59)	15	0.032
Palpitations	17.38	31.31	0.000	4 (2–7)	1	15	5.03 (±4.02)	1,2	6 (3–9)	1	15	6.39 (±4.15)	1,5	0.004
Ophthalmalgia	17.38	27.81	0.000	5 (3–7)	1	15	5.57 (±3.41)	5	5 (3–7)	1	15	5.42 (±3.60)	3	0.574
Diaphoresis	16.62	22.34	0.014	4 (2–5)	1	15	4.55 (±3.33)	4	5 (3–9)	1	15	5.93 (±4.00)	3	0.011
Shoulder pain	16.12	19.85	0.095	6 (3–8)	1	15	5.80 (±3.57)	3	5 (3–8)	1	15	6.05 (±3.94)	3	0.861
Hoarseness	15.87	20.15	0.057	4 (2–8)	1	15	5.67 (±4.69)	1	5 (2–9)	1	15	6.05 (±4.36)	1	0.294
Sneezing	15.37	21.17	0.011	3 (2–5)	1	15	4.46 (±3.74)	1	4 (2–7)	1	15	5.27 (±3.74)	3	0.050
Skin hyperaesthesia	15.11	19.2	0.064	5 (3–7.5)	1	13	5.25 (±3.48)	1,3	5 (3–7)	1	15	5.64 (±3.50)	3,4	0.370
Abdominal pain/ache	13.6	21.39	0.001	3 (2–5)	1	15	4.06 (±3.30)	2,3	4 (2–6)	1	15	4.71 (±3.69)	1	0.219
Metallic taste	12.85	16.13	0.110	5 (3–9)	1	15	6.24 (±4.27)	1,3	5 (3–8)	1	15	5.91 (±3.89)	1	0.718
Nausea	11.34	23.36	0.000	3 (1–6)	1	14	3.93 (±3.47)	1	5 (2–8)	1	15	5.45 (±3.79)	1,2,3	0.003
Limb numbness	10.08	13.5	0.071	3 (1–8)	1	15	5.13 (±4.74)	1	4 (2–8)	1	15	5.42 (±4.19)	1	0.338
Polydipsia	9.57	19.71	0.000	7 (4–10)	1	15	6.92 (±3.81)	4,6,8,10	8 (5–12)	1	15	8.21 (±4.38)	15	0.095
Skin lesions	9.07	11.02	0.265	4 (1–6.5)	1	15	4.56 (±3.97)	1	4 (2–7)	1	15	4.88 (±3.95)	1	0.510
Conjunctivitis	8.82	11.02	0.194	4 (2–7)	1	14	4.60 (±3.44)	1	5 (2.5–7.5)	1	15	5.49 (±4.10)	1	0.279
Blood pressure elevation	8.06	9.93	0.264	7 (3–9)	1	15	6.84 (±4.11)	3	4 (3–8)	1	15	5.76 (±4.13)	3	0.144
Odontalgia	6.80	8.18	0.371	4 (1–6)	1	15	4.37 (±3.97)	1	4 (2–6)	1	15	4.54 (±3.38)	1	0.496
Ear ache/pain	6.30	10.66	0.010	3 (1–4)	1	12	3.36 (±3.08)	1	3 (1–5)	1	15	3.70 (±3.20)	1	0.467
Blood pressure reduction	4.79	5.84	0.422	6 (1–8)	1	11	5.37 (±3.65)	1	6 (3–10)	1	15	6.56 (±4.46)	1	0.394
Alopecia	4.79	18.61	0.000	3 (1–5)	1	15	3.84 (±3.73)	1	5 (1–8)	1	15	5.42 (±4.41)	1	0.104
Vomiting	4.28	7.88	0.014	1 (1–3)	1	4	1.82 (±1.24)	1	2 (1–4)	1	13	2.97 (±2.53)	1	0.038
Hemoptysis	4.28	1.46	0.001	2 (2–5)	1	9	3.00 (±2.12)	2	2 (1–5)	1	13	3.45 (±3.47)	1	0.582
Aphonia	2.77	2.63	0.876	2 (1–5)	1	11	3.36 (±3.17)	1	3 (1–5)	1	14	3.58 (±3.19)	1	0.825
Toes discoloration	2.77	1.97	0.333	3 (1–10)	1	14	5.36 (±5.01)	1	2 (1–7)	1	14	4.11 (±3.75)	1	0.702
Urinary incontinence	2.77	3.65	0.398	2 (1–5)	1	15	4.18 (±4.45)	1,2	6 (2–10)	1	14	6.06 (±4.25)	1	0.179

1 Duration of the symptom in days in men [median (LQ-UQ)].

2 Duration of the symptom in days in women [median (LQ-UQ)].

3 Duration of the symptom in days. Average (± SD).

Statistic correlation counted with chi2.

Seven symptoms occurred in over 50% of participating men: fatigue (86%), subfebrility (70%), anosmia (65%), myalgias (56%), headache (55%), fever (55%) and cough (53%). Nine symptoms were declared by over 50% of women: fatigue (88%), anosmia (76%), headache (74%), subfebrility (64%), myalgias (62%), ageusia (59%), cough (53%), lack of appetite (52%) and back pain (52%).

Regarding the duration of COVID-19 symptoms 12 lasted significantly longer in women than in men: hypothermia, fatigue, sneezing, nausea, vomiting, lack of appetite, hyperhidrosis, diaphoresis, palpitations, insomnia, hypersomnia and xerostomia. The minimum duration of each symptom was 1 day, maximum was ‘15 days or more’. Only 21 participants (1.19%) chose ‘no symptoms’ in the survey: 2.02% of responding women and 0.95% of responding man. The difference was not statistically significant (*p* = 0.084).

## 5. Discussion

Despite the growing knowledge of the SARS-CoV-2 virus acquired since the end of 2019, its symptoms are not well recognized and a full list is still being researched. As the symptoms of COVID-19 are mostly very similar to those of other infectious diseases, it is problematic to differentiate the condition from other respiratory tract diseases (34). It may be hard to tell the difference between novel coronavirus infection and common cold or influenza, especially in primary care where diagnostic procedures are limited and number of cases is significant, especially in infectious seasons. Hence, there is a need for a better understanding of the symptoms, their occurrence in time and their relationship with patient sex. Knowing the prevalence of COVID-19 symptoms in a single population will help diagnose COVID-19 on many levels. Knowing the specific combination of symptoms that are most likely to occur in COVID-19 will make it easier to isolate COVID-19 patients from a group of similar infectious diseases at the clinical stage, especially in primary care. It will also help in screening patients who need to be diagnosed further, e.g., using antigen tests. Better knowledge of the symptoms characteristic of a given disease is associated with easier recognition at the stage of the patient, and therefore easier self-isolation and better control of the spread of this disease, thus being highly clinically and epidemiologically important.

In the studied population, the most prevalent symptoms of COVID-19 infection were fatigue, anosmia, headache, subfebrility and myalgia; these were reported by 61–88% participants. These findings are different from those obtained in other studies of COVID-19 symptoms. For example, one of the first publications summarizing the findings associated with hospitalized COVID-19 patients found cough to be the most common symptom (presented by 67.8% patients) followed by fatigue (38.1% patients) (9). Amongst the population in our study, cough was not that prevalent, being the seventh most common symptom (53%). This difference could be due to the fact that Guan’s study was based mostly on hospitalized patients (93.6%) while only 4.7% were admitted to the hospital in the present study (9).

In general the prevalence of COVID-19 symptoms differ depending on the studied population. For example, in an Iranian study of 23,749 patients from 74 hospitals in Teheran, the most prevalent symptoms were cough (50.51%), respiratory distress (43.55%), muscular pain or fatigue (38.94%), gastrointestinal problems (10.4%) and headache (4.72%) (35). A Chinese meta-analysis of 38 studies with 3,062 patients found these to be fever (80.4%), cough (63.1%) and fatigue (46.0%) but not anosmia or ageusia (36). Similar findings were described in meta-analysis of 54 mainly Chinese publications, where fever (81.2%), cough (58.5%) and fatigue (38.5%) were listed as the most prevalent symptoms (10). Other Chinese meta-analyses listed fever, fatigue and cough as the most common symptoms (37, 38). In our population, fever was ranked 12 among the most common presentations; however, a subfebrility, i.e., a high temperature, was reported by nearly twice as many survey participants as fever (1,154 vs. 768).

Similarly, fever (73.5%) and cough (61.0%) were found to be the most common symptoms in an Egyptian review of 1773 COVID-19 patients (39). A meta-analysis by Ghayda et al. found the most common symptoms to be fever (prevalence - 77%), cough (60%) and fatigue/myalgia (31%); the authors also reported gastrointestinal symptoms including diarrhea (6%) and nausea/vomiting (5%) (40), which were much more common in the present study: 29% for diarrhea, 21% for nausea and 7% for vomiting.

In some cases, our present findings were quite divergent from those of previous studies. In one meta-analysis, the most common symptoms were found to be fever (97%), cough (70%) and myalgia/fatigue (39%), while our respective findings were fever/cough 43%/53% and myalgia/fatigue 61%/88%, also diarrhea was less prevalent than in our study (8% vs. 29%) (41). Another meta-analysis found the pooled prevalence of loss of appetite, nausea/vomiting, diarrhea and abdominal pain/discomfort to be 27, 10, 12 and 9%, respectively (42), compared to 50, 28, 29 and 20% in our study. These differences may be due to variation in populations, regions or SARS-CoV-2 variants among others. In addition, our study mostly includes outpatient subjects, which may also affect the results, since other studies rely on inpatients data mostly. We may only suspect that there may be a correlation between the patient presentation and his further path in the health care system. For example, COVID-19 patients with muscle pain and digestive symptoms are less likely to be hospitalized than those with fever and cough, however this needs further investigation.

Nonetheless, SARS-CoV-2 infection presents with variety of symptoms from different body systems. This is most likely the effect of the wide range of angiotensin converting enzyme 2 (ACE2) expression in human tissues, with the small intestine, testis, kidneys and heart being the highest (43). ACE2 acts to degrade angiotensin II into angiotensin (1–7) which ultimately leads to a decrease in blood pressure (44). Symptoms believed to be specific for COVID-19, i.e., anosmia and ageusia, were not that prevalent the first day (15.27 and 11.93% respectively), but were reported by more than 40% of patients on day five and day six, respectively, (Figure 4). The prevalence of olfactory dysfunctions has been found to range from 5.6 to 98% of COVID-19 patients, with differences between China/Asia and Europe/USA (45), probably due to different expression of the ACE2 receptor in global populations (46). Although they may precede, follow or co-occur with other general symptoms (47), these symptoms were present in more than 50% of participants in the present study, starting from day 5. This suggests that in order to differentiate COVID-19 in the first days of the disease, the patient should be asked about fatigue, rather than anosmia, ageusia or nasal discharge at the first visit, especially in primary care. These symptoms are more common than anosmia or ageusia in the first days of COVID-19. Olfactory and gustatory problems become more prevalent starting from day 5.

**Figure 5:**
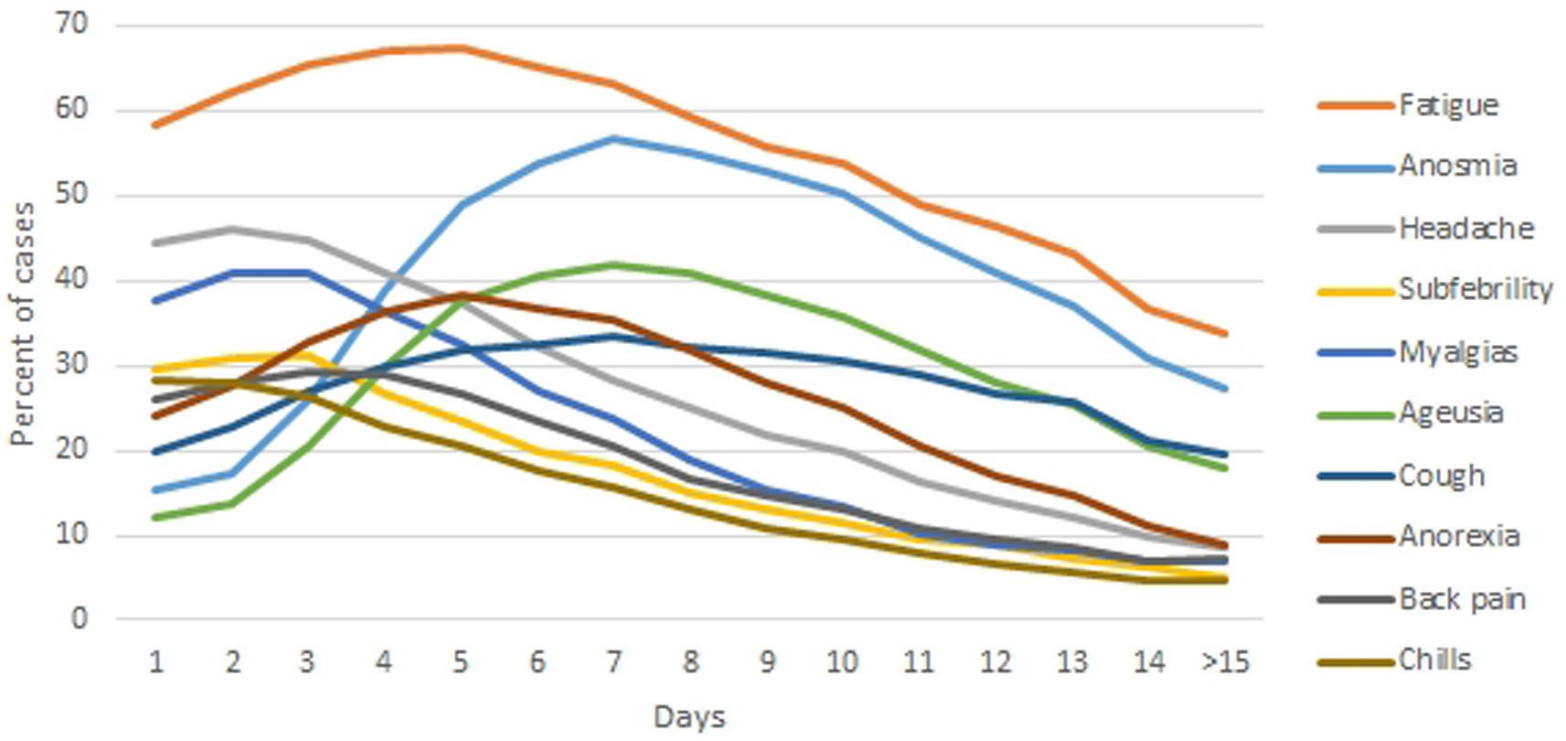
The course of the 10 most commonly reported symptoms of COVID-19 in the first 14 days of the disease, as reported by survey participants [n = 2,408].

Our study confirmed previous findings that anosmia and ageusia are more frequent COVID-19 dysfunctions in women than men (47–50). This may be due to the fact that activation of the Toll-like receptor (TLR), a protein responsible for pathogen recognition in the human innate immune system (51), is related to X chromosomes; this may lead to various inflammatory conditions and clinical courses in both sexes (52). No statistically significant differences in the duration of anosmia or ageusia were observed between men and women; in both cases, the mean duration of symptoms was eight days, which is consistent with the findings of other authors (45). Because smell and taste disorders are more prominent in COVID-19 than other seasonal viral diseases (e.g., influenza (53)) it is of high importance for physicians to recognize it as one of the most common and major symptoms of COVID-19.

The longest-lasting symptoms were fatigue, cough, nasal dryness, xerostomia and polydipsia (15 days or more). Although our study focuses on the first two weeks of COVID-19 infection, these six persisting symptoms lasted over 14 days, may be assumed to be prolonged and be a part of condition known as *long COVID* (54). Although prolonged illness is well documented in hospitalized patients (55), it also affects individuals with mild infection who do not need hospitalization (56). Neither nasal dryness, xerostomia or polydipsia were not included in a list of 48 clinical symptoms given by a previous meta-analysis (57). Thanks to our study, these symptoms may be added to the rich symptomatology of novel coronavirus infection. Lopez-Leon et al. found study fatigue and headache to be the most prevalent symptoms of long-COVID (80 and 58% respectively), similar to our study; however, attention disorder was also listed in third place (27%) (57). In the present study, the highest ranking psychiatric symptom was depression, which was ranked 21 with a similar prevalence (30%). It is possible that, in our population, psychiatric problems were not the most common within the first two weeks of COVID-19, but could become more apparent over the course of longer-term infection as other symptoms subside. Similarly, alopecia was also not that prevalent in our study (15.5%) compared to Lopez-Leon (25%) (57).

Our findings suggest that women suffering from COVID-19 are more symptomatic within the first 14 days of the disease and that many of their symptoms persist longer than for men. Many sex-dependent factors affecting COVID-19 have been confirmed previously. A large cohort study of 17 million patients in England found a hazard ratio of 1.59 for higher risk of death due to COVID-19 in men (58). Higher ratio of male-to-female death was also confirmed in France, Spain, Italy, Switzerland, Germany and China (59). Although the cause of these differences is currently unknown, it is thought that differences in sex hormones may contribute to different immunologic responses between the sexes (60). Takahashi et al. report that cytokines IL-8, IL-18, CCL5 were significantly elevated in male COVID-19 patients, and that CD38 and HLA-DR-positive activated T cells in female COVID-19 patients (61). Meng et al. found significant differences between male and female COVID-19 patients in eleven laboratory parameters including glucose, CRP and creatinine concentration (62).

ACE2 is more strongly expressed in men than women, particularly under pathological conditions (63), its tissue expression is also higher in men than women (64); moreover, angiotensin II receptor type 1 (AT1R; which SARS-CoV-2 binds to) is downregulated by estrogens (65). In addition to AT1R, the expression of TMPRSS2, a protein that primes SARS-CoV-2 entry into cells, is upregulated by androgens (66). Low levels of baseline serum testosterone in men may also contribute to increased cardiovascular risk in COVID-19 (67). This hormone also affects cytokine production (e.g., suppresses IL-6, IL-1beta and TNF-alpha, enhances production of IL-10) (68), suppresses T-helper cells, enhances regulatory T-cell differentiation (69), and reduces B-cell proliferation and humoral response (70). Moreover men with lower testosterone levels are predisposed to pulmonary and systematic inflammation and develop worse general parameters (67). The response to pathogens also differs between sexes: adult women demonstrate twice the protective antibody response after vaccination against various viral diseases compared with men (71).

Our findings confirm previous observations that sex affects the COVID-19 presentation. Sex-based differences were also found during the MERS outbreak in 2013–2014, with the fatality rate being 52% in men, while in women it was 23% (72). Other factors may also be involved in these observed differences: for example, the presence of comorbidities such as hypertension, cardiovascular disease, chronic obstructive pulmonary disease, which are more prevalent in men than women (73). These may also be caused by higher smoking and alcohol consumption observed in men (74, 75). In Poland, although 54.3% of COVID-19 cases were confirmed in women, 56.5% of COVID-19 deaths were confirmed in men, which also supports the thesis of gender differences in COVID-19 presentation (76). Nonetheless, the evidence for influence of patient’s sex on COVID-19 course is increasing and our study also supports this thesis with its findings.

### 5.1. Strengths and limitations

The strength of our study not mentioned earlier is the nature of its population: it included over 2000 patients with diagnosed COVID-19. Out of 7,034 participants who started the survey, 36.7% were disqualified for analysis due to lack of testing for SARS-CoV-2. It is possible that many of the respondents were COVID-19 convalescents, as the survey announcements were targeted at this group; if so, the high disqualification rate suggests that vast number of patients were convinced that they had been suffering from COVID-19 without any medical evidence. As antigen tests have only been available in Poland since May 2020 (77), it was assumed that the patients claiming a positive test for SARS-CoV-2 would have received an RT-PCR test. Therefore, only participants with a positive PCR test were included: those who had undergone antigen tests, with positive COVID-19 antibodies in blood serum, or those who had suffered from non-COVID related infection, were not included in the final analysis. Restricting inclusion to only PCR-positive patients improves the reliability of the symptoms given by patients and limits the results of the study to true positive COVID-19 patients.

The major limitation of the present study is that it was not possible to verify clinically the symptoms provided by survey participants. The survey was conducted online and subjects reported the symptoms based on their subjective recognition: the study was anonymous to encourage the participants to present honest answers. What is more IP addresses where not collected due to ethical reasons (identification of participants). Indeed, the instructions on the first screen emphasized the need to provide ‘sincere and true answers’ together with an explanation. The table of symptoms was also large (55 options of symptoms in rows and 15 day options in the columns, making 825 options), although this may discourage anyone whose aim was other than to share real data about his illness. In addition, the survey included 20 questions on nine screens, and no incentive was offered for completion; as such, a high level of motivation and persistence was needed to finish, and the respondent was less likely to provide intentionally false answers. Out of 7,034 who started the survey 66% were not qualified to the final analysis due to exclusion criteria, which shows our strict approach to include only valuable data. Moreover this was a cross-sectional survey conducted on specific sample of Polish population. Although important correlations were found, generalization of our findings is not entirely possible and needs additional research.

Although COVID-19 may be asymptomatic (45) only 2% of men and 1% of women in our study declared that they had no symptoms. It is possible that it was because of the sample bias - only those who suffered from COVID-19 symptoms were willing to take part.

Although the alpha SARS-CoV-2 variant was on the rise during data collection (Figure 1) we would rather not connect all the listed symptoms with this particular variant. According to ECDC data, the percentage of sequenced cases in Poland ranged from 0.2% in week 52–2020 to 1% in week 08–2021 (32). Hence, we are reluctant to confirm any link between reported symptoms and the alpha variant; however, we may assume that the symptoms presented in this publication refer to alpha variant. Even so, more studies sequencing the COVID-19 variants and collecting presented symptoms are needed to identify characteristic disease presentations and any symptomatic differences between variants.

## 6. Conclusion

COVID-19 research on its symptomatology is evolving. Our study have found that out of 54 identified symptoms occurring during the first 14 days of infection, the most common were fatigue, anosmia and headache. Symptom presentation was also found to differ between the sexes, shedding new light on the nature of COVID-19 presentation in men and women.

Although studies have summarized the symptoms of long COVID-19, there is still lack of good quality data concerning the symptoms of acute SARS-CoV-2 infection. In response, our findings describe not only a broad symptomatology of acute COVID-19 but also its dependence on sex. The full clinical profile of COVID-19 cannot be limited to a few major symptoms. Future studies are therefore needed to examine its presentation against the background of other factors such as COVID variant, geographical location or population type.

## Data availability statement

The raw data supporting the conclusions of this article will be made available by the authors, without undue reservation.

## Ethics statement

The studies involving human participants were reviewed and approved by the Bioethical Commission of the Medical University of Lodz, decision number RNN/319/20/KE, December 15, 2020. Written informed consent for participation was not required for this study in accordance with the national legislation and the institutional requirements.

## Author contributions

PL conceived and planned the study and collected the data. PL and PK designed the questionnaire and contributed to the writing of the manuscript. IB designed the figures and contributed to the interpretation of the results and to the discussion. KW worked on the manuscript and contributed to the interpretation of the results. JL performed statistical analysis and contributed to the interpretation of the obtained results. PK contributed to planning the study and supervised the project. All authors discussed the results and contributed to the final manuscript.

## Conflict of interest

The authors declare that the research was conducted in the absence of any commercial or financial relationships that could be construed as a potential conflict of interest.

## Publisher’s note

All claims expressed in this article are solely those of the authors and do not necessarily represent those of their affiliated organizations, or those of the publisher, the editors and the reviewers. Any product that may be evaluated in this article, or claim that may be made by its manufacturer, is not guaranteed or endorsed by the publisher.

## Abbreviations

Avg: average
COVID-19: coronavirus disease 2019
ECDC: European Centre for Disease Prevention and Control
LQ: lower quartile
Min: minimum
Max: maximum
SARS-CoV-2: severe acute respiratory syndrome coronavirus 2
SD: standard deviation
UQ: upper quartile
