# Supplementary material for: The prevalence of symptoms and its correlation with sex in polish COVID-19 adult patients: Cross-sectional online open survey
Source: Front Med (Lausanne). 2023 Apr 5;10:1121558. doi: 10.3389/fmed.2023.1121558 (PMC10113468; doi:10.3389/fmed.2023.1121558)
Supplement: Supplementary file 1 [file Table_1.DOCX]

Supplementary file

Document 1. Facebook groups and news web sites devoted to the topic of COVID-19 where the link to the survey was shared.

Facebook groups:

COVID-19 - Koronawirus - grupa wsparcia dla pozytywnych i ozdrowieńców. (Eng. COVID-19 Coronavirus - Support group for positives and convalescents.)

Covid-19 - relacje chorych (wymiana doświadczeń) (ang. COVID-19 - experience exchange of sick)

Koronawirus Lekarze (Eng. Coronavirus physicians)

Covid-19 Doctors

Koronawirus COVID-19 ŁÓDŹ - INFORMACJE (Eng. Coronavirus COVID-19 Lodz - Information)

Web sites:

https://wydarzenia.interia.pl/raporty/raport-koronawirus-chiny/aktualnosci/news-koronawirus-premier-boris-johnson-oglosil-ogolnokrajowy-lock,nId,4966651

https://wydarzenia.interia.pl/raporty/raport-koronawirus-chiny/aktualnosci/news-wielka-brytania-rekordowa-liczba-nowych-zakazen-koronawiruse,nId,4966644

https://wydarzenia.interia.pl/raporty/raport-koronawirus-chiny/aktualnosci/news-odpornosc-na-covid-19-najnowsze-wyniki-badan,nId,4970085

https://www.o2.pl/informacje/who-zaszczepieni-tez-powinni-przechodzic-kwarantanne-podali-powod-6591575767604000a

https://www.o2.pl/informacje/koronawirus-nowe-informacje-ws-szczepionki-na-covid-19-od-moderny-6594312537275200a

https://portal.abczdrowie.pl/szczepsieniepanikuj-polacy-boja-sie-igiel-prof-simon-lekarze-tez-sie-boja-ale-to-nie-powod-zeby-sie-nie-szczepic#comments

https://www.o2.pl/informacje/prosze-popatrzec-na-polske-mocne-slowa-bylego-kanclerza-niemiec-6597934587595648a

https://www.o2.pl/zdrowie/ekspert-ostrzega-przed-nowa-pandemia-moze-nas-dopasc-podczas-lockdownu-6598160398834656a

https://www.o2.pl/sport/to-moze-byc-rewolucja-nowy-pomysl-na-gre-w-czasach-pandemii-6599334908074880a

https://www.o2.pl/informacje/koronawirus-chiny-tak-zle-nie-bylo-od-marca-6600929685015424a

https://www.o2.pl/zdrowie/zabronili-maseczek-z-tkanin-w-komunikacji-miejskiej-czeka-nas-to-samo-6601028256212609a

https://wydarzenia.interia.pl/raporty/raport-koronawirus-chiny/aktualnosci/news-pozytywne-informacje-z-izraela-na-temat-szczepionki-pfizer,nId,5008863

https://wydarzenia.interia.pl/raporty/raport-koronawirus-chiny/aktualnosci/news-who-zaadaptujemy-szczepionki-do-nowych-wariantow-koronawirus,nId,5013242

https://wydarzenia.interia.pl/raporty/raport-priorytety-ue-w-dobie-pandemii/aktualnosci/news-ciemnoczerwone-strefy-zakazen-nowe-srodki-dotyczace-pandemii,nId,5023478

https://wydarzenia.interia.pl/swiat/news-szczepienia-w-wielkiej-brytanii-37-proc-zaszczepionych-mialo,nId,5029897

https://wydarzenia.interia.pl/raporty/raport-koronawirus-chiny/polska/news-koronawirus-w-polsce-raport-ministerstwa-zdrowia-z-13-lutego,nId,5047612

https://wiadomosci.wp.pl/koronawirus-w-polsce-dworczyk-i-niedzielski-6609885858667392a

https://www.money.pl/gospodarka/koronawirus-w-polsce-przyspiesza-w-niemczech-hamuje-kolejne-ograniczenia-coraz-blizej-6609865699457888a.html

https://www.o2.pl/informacje/koronawirus-w-polsce-nadchodzi-powazna-zmiana-niektore-maseczki-pojda-do-kosza-6609796231986016a

https://www.o2.pl/informacje/smiertelnosc-wirusa-nipah-to-75-proc-who-ostrzega-przed-nowa-pandemia-6610235037531008a

https://www.o2.pl/informacje/polski-morderca-koronawirusa-tak-twierdza-amerykanie-6611766919932864a

https://wiadomosci.wp.pl/koronawirus-czy-pod-koniec-maja-odpoczniemy-od-pandemii-szef-biontech-odpowiada-6612798960192480a

https://wydarzenia.interia.pl/polska/news-sopot-w-strefie-zagrozenia-wystapienia-ptasiej-grypy,nId,5076318
