# Supplementary material for: The prevalence of symptoms and its correlation with sex in polish COVID-19 adult patients: Cross-sectional online open survey
Source: Front Med (Lausanne). 2023 Apr 5;10:1121558. doi: 10.3389/fmed.2023.1121558 (PMC10113468; doi:10.3389/fmed.2023.1121558)
Supplement: Supplementary file 2 [file Table_2.DOCX]

Document 2. Announcement presented on websites listed in Document 1.

Polish original

Pomóż lekarzom zrozumieć COVID-19. Jeśli przechorowałeś/aś tę chorobę wypełnij 3-minutową anonimową ankietę na objawycovid(kropka)pl aby pomóc naukowcom z Uniwersytetu Medycznego w Łodzi rozpoznawać jej objawy i lepiej leczyć pacjentów.

English translation

Help physicians understand COVID-19. If you have suffered from this disease, complete a 3-minute anonymous survey at objawycovid(dot)pl to help scientists from the Medical University of Lodz diagnose symptoms and better treat patients.
